# Physical Fitness Is Directly Related to Exercise Capacity and Ventilatory Response to Exercise in Men with HFrEF

**DOI:** 10.3390/jcm13123465

**Published:** 2024-06-13

**Authors:** Olga Kisiel-Sekura, Magdalena Wójciak, Agnieszka Siennicka, Michał Tkaczyszyn, Marcin Drozd, Ewa A. Jankowska, Adrian Doroszko, Waldemar Banasiak, Kinga Węgrzynowska-Teodorczyk

**Affiliations:** 1 Centre for Heart Diseases, 4th Military Hospital, 50-981 Wroclaw, Poland; 2 Department of Physiology, Wroclaw Medical University, 50-367 Wroclaw, Poland; 3 Institute of Heart Diseases, Wroclaw Medical University, 50-367 Wroclaw, Poland; 4 Institute of Heart Diseases, University Hospital, 50-556 Wroclaw, Poland; 5 Faculty of Medicine, Wroclaw University of Science and Technology, 51-377 Wroclaw, Poland; 6 Faculty of Physiotherapy, Wroclaw University of Health and Sport Sciences, 51-612 Wroclaw, Poland

**Keywords:** HFrEF, physical fitness, exercise capacity, cardiopulmonary exercise testing, ventilatory response, physical therapy

## Abstract

**Background**: Heart failure (HF) patients experience reduced functional fitness level (determining the performance of routine, daily activities) and diminished exercise capacity (linked to more effortful activities). **Aim**: The aim of the study is to assess this relationship using functional fitness tests compared to peak VO2 and VE/VCO2 slope in the context of exercise capacity and ventilatory response to exercise. **Methods**: A total of 382 men with stable HFrEF (age: 61 ± 10, NYHA class I/II/III/IV: 16/50/32/2%, LVEF: 30.5 ± 8.3%) underwent cardiopulmonary exercise testing (CPX) and a Senior Fitness Test (SFT). Afterwards, the patients were divided according to the 2capacity with peak VO2 ≥ 18 mL/kg/min, those with higher or lower ventilatory responses (VE/VCO2 slope ≥ 35 vs. <35) to the exercise were compared. **Results**: Patients who covered shorter distances in the 6 min walking test showed worse results in the functional tests (‘stand up and go’, ‘chair stand’ and ‘arm curl’) and CPX (lower peak VO2, shorter exercise time and higher VE/VCO2 slope). Subjects classified into Class D demonstrated the worst results in all elements of SFT; those in Class A demonstrated the best results. Significant differences that were analogous occurred also between classes B and C. Among the participants who reached peak VO2 ≥ 18 mL/kg/min (n = 170), those with VE/VCO2 slope ≥ 35 were characterized by worse physical fitness as compared to those with VE/VCO2 < 35. **Conclusion**: Reduced exercise tolerance led to worsening physical function in patients with HFrEF. Moreover, limitations in physical fitness seem to be distinctive for those patients showing excessive ventilatory response to exercise slope VE/VCO2 (≥35). The Senior Fitness Test may be considered as a useful tool for assessing comprehensive functional and clinical status and risk stratification in patients with HFrEF, especially those with extremely low exercise capacity.

## 1. Introduction

According to new guidelines, heart failure (HF) is defined as a clinical syndrome of symptoms and signs. Including left ventricular ejection fraction (LVEF) and cardiac structural and/or functional abnormalities, HF is classified into distinct groups: heart failure with reduced ejection fraction (HFrEF), heart failure with mildly reduced ejection fraction (HFmrEF), and heart failure with preserved ejection fraction (HFpEF). Patients with HF are limited in their ability to tolerate physical effort due to abnormal, excessive responses, i.e., early occurrence of dyspnoea and fatigue, which constitute the main clinical symptoms of HF [1,2,3,4].

**Figure 1:**
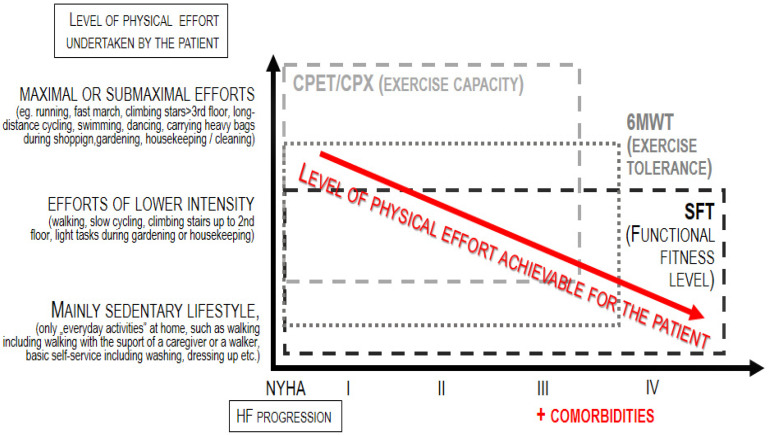
Application of physiotherapeutic tests according to the progression of heart failure.

HF patients experience both diminished exercise capacity (linked to situations when the effort is more intensive or even maximal) and—as the disease becomes more advanced —also reduced physical fitness level (associated with the functional performance of efforts of low intensity, corresponding to the routine, daily activities) [5,6]. This is due to frailty syndrome, long and frequent hospitalization, bed rest, fewer activities and more episodes of decompensation. As a consequence, patients lose the independence and autonomy that ensure their quality of life, psychophysical and health well-being. As shown in Figure 1, exercise capacity refers to maximal or submaximal efforts; thus, the test dedicated for the assessment of exercise capacity, i.e., the cardiopulmonary exercise test (CPX), can be applied only to patients who are able to achieve this level of effort [7]. On the other hand, the term fitness refers to the basic level of independent everyday functioning (such as self-dressing); thus, tests dedicated to measuring the level of physical fitness can be applied to both patients who can perform maximal/submaximal efforts as well as patients whose everyday functioning is significantly limited–like, for instance, a huge part of the population of HF patients [8,9].

The only way to assess how these patients tolerate physical effort is to measure the fitness level instead of the exercise capacity.

Therefore, the physiotherapeutic evaluation of patients with HF should be based on both the measures of exercise capacity as well as measures of physical fitness [6,10,11].

CPX is a ‘gold standard’ in an objective evaluation of exercise capacity [7] based on the measurement of time of exercise, peak oxygen uptake (peak VO2) as well as ventilatory response to exercise. Exercise ventilation insufficiency is a hallmark manifestation of HF. An index of ventilatory response to exercise, the VE/VCO2 slope, is calculated as the slope of the linear relationship between minute ventilation and carbon dioxide production [12].

The peak VO2 and the VE/VCO2 slope have been shown to be strong prognostic parameters of the results of cardiac rehabilitation, reduction in the patient’s quality of life, cardiac-related hospitalization, left ventricular assist device implantation, heart transplantation and mortality in patients with HF [13,14,15,16,17].

The Senior Fitness Test [18] for assessing functional fitness among older adults reflects the level of impairment of the functional status during daily activities. In our previous studies, we applied a Senior Fitness Test to assess the physical fitness of men with HFrEF. We observed that a decreased level of aerobic endurance, agility, dynamic balance and muscular endurance occurred in patients with more advanced HF, reflected by a higher NYHA class, higher level of NT-proBNP, presence of anaemia and impaired renal function [6].

Until now, the relation between physical fitness and exercise capacity has not been comprehensively investigated in patients with HF.

### Objective

The null hypothesis states that physical fitness levels, evaluated during simple functional fitness tests, do not depend on physical capacity derived from CPX, reflecting the patient’s exercise capacity.

Additionally, more excessive work of the respiratory muscles assessed by the VE/VCO2 slope does not depend on physical fitness.

## 2. Materials and Methods

### 2.1. Study Subjects

Subjects were recruited among patients admitted to the Heart Failure Outpatient Clinic within the Centre for Heart Diseases, Military Hospital in Wroclaw.

**Figure 2:**
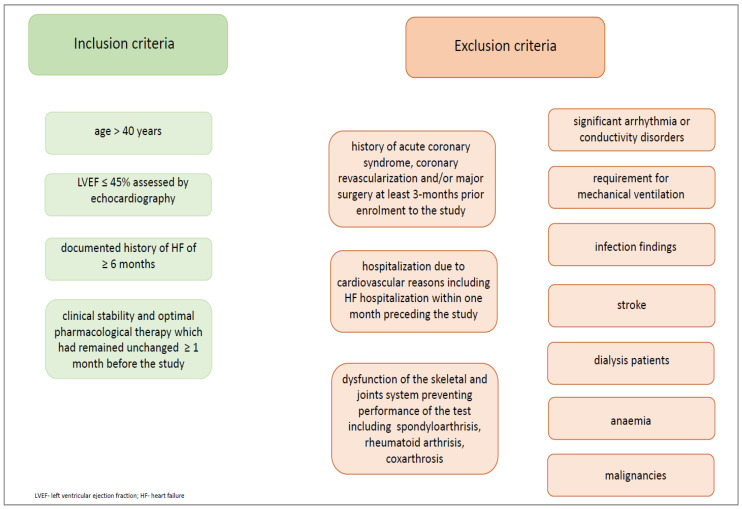
Inclusion and exclusion criteria.

We recruited only men with HFrEF. Patients were selected based on the following inclusion and exclusion criteria (Figure 2.)

### 2.2. Methods

Maximal symptom-limited CPX was performed using the modified Bruce protocol (Marquette Series GE T2000 Treadmill; GE Medical Systems, Milwaukee, WI, USA). Patients were encouraged to perform the exercise until exhaustion. Before each test, the equipment (including sensors of oxygen and carbon dioxide concentration and flow) was calibrated. A standard 12-lead electrocardiogram and a heart rate were recorded during the 5 min rest, and during the exercise and 5 min recovery phase. Blood pressure was measured manually at rest, at the end of each exercise stage, at peak exercise and during recovery using a sphygmomanometer.

Oxygen uptake (VO2 in L/min), carbon dioxide production (VCO2 in L/min) and minute ventilation (VE in L/min), were measured breath by breath, using the MedGraphics Ultima Series Cardiorespiratory Diagnostics gas analyser (MGC Diagnostic Corporation, Minnesota, USA) [19]. The peak VO2 was averaged from three-second intervals during the last 20 s of exercise. This calculation was also used to divide the examined patients according to the Weber functional classification for HF [20], which assigns the deterioration of functional capacity to a particular class defined as follows:

- A class: peak VO2 > 20 mL/kg/min (mild or absent deterioration),
- B class: peak VO2 16.1–20 mL/kg/min (mild or moderate deterioration),
- C class: peak VO2 10–16 mL/kg/min (moderate-severe deterioration) and
- D class: peak VO2 < 10 mL/kg/min (severe deterioration).

Minute ventilation and VCO2 data, obtained from the period between exercise onset and peak of exercise, were imported to Microsoft Excel for the calculation of the VE/VCO2 slope using linear regression (y = mx + b, m = slope). Values of peak VO2 and the VE/VCO2 slope were used to conduct a sub-analysis within a group of patients with preserved exercise capacity (i.e., peak VO2 ≥ 18 mL/kg/min) focusing on the intensity of ventilatory response to exercise (reflected by either a VE/VCO2 slope < 35 or a ≥35 VE/VCO2 slope) [19].

### 2.3. The Functional Fitness Test (The Senior Fitness Test)

The Senior Fitness Test proposed by Rikki and Jones [18] was performed to assess the level of physical fitness. The usefulness and good reproducibility of this test in patients with HF was confirmed in our previous study [21].

The Senior Fitness Test is composed of the following six motor tasks (Table 1).

Each of the test tasks were in detail described in words, then demonstrated by a researcher. According to the safety rules of cardiac rehabilitation, blood pressure and heart rate were measured before we started the Senior Fitness Test and after chair stand and 6MWT tasks.

All tests were performed on the same day; the CPX was used after the Senior Fitness Test, following at least two hours of resting.

### 2.4. Statistical Analysis

We used the Shapiro–Wilk test to assess for the normality of distribution. Continuous variables with a normal distribution and homogenic variance [e.g., age, body mass index (BMI) or left ventricular ejection fraction (LVEF)] were presented as means (x) ± standard deviations (SD). Continuous variables with a skewed distribution (e.g., plasma NT-pro BNP) were expressed as medians with lower and upper quartiles. The categorical variables were expressed as numbers with percentages. The relationship between the tasks of the Senior Fitness Test and CPX data was tested using the Spearman correlation coefficient. Analysis of the inter-group differences were based on the Mann–Whitney U-test or the one-way analysis of variance ANOVA with post-hoc comparison Tukey test, where appropriate. A value of *p* < 0.05 was considered statistically significant.

## 3. Results

A group of 382 men with stable HFrEF were included in the study. Baseline clinical characteristics of all the studied patients (and selected in Weber class) and CPX test results are presented in Table 2.

All participants were able to perform both the Senior Fitness Test and the CPX test. The Senior Fitness Test was well tolerated by all patients, which means that there were no life- or health-threatening incidents during or immediately after the test.

During the Senior Fitness test patients (n = 382) achieved the following results in each tasks:

- 6MWT [m] 480.0 (415.0–547.0),
- stand up and go [ s] 5.5 (4.8–6.4),
- chair stand [number] 14.0 (12.0–17.0),
- arm curl [number] 15.0 (13.0–18.0),
- chair sit and reach [cm] 0.0 (−8.0–5.0),
- back scratch [cm] −8.0 (−16.0–0.0).

Table 3 summarizes the results of analysis of the relationships between level of physical fitness assessed by Senior Fitness Test and exercise capacity exhibited by parameters from CPX. Patients who covered shorter distance during the 6MWT needed a longer time for completing the ‘stand up and go’ test. These patients also performed a lower number of repetitions during both the ‘chair stand’ and ‘arm curl’ tasks; they had lower peak VO2, shorter exercise times and a higher VE/VCO2 slope during the CPX. Essentially, only flexibility parameters showed no relations with exercise capacity.

Worse muscle endurance, agility and dynamic balance corelate with lower exercise tolerance.

We observed differences in the level of physical fitness depending on Weber’s classification (Table 4). Patients classified into Class D demonstrated the worst results in all elements of the Senior Fitness Test. Accordingly, patients allocated to Class A demonstrated the best results. The worsening of exercise tolerance, muscle endurance and agility corresponds to lower peak VO2 in the appropriate Weber’s class. Furthermore, significant differences occurred between each Weber’s class.

Table 5 demonstrates the results of final analysis conducted within a subgroup of participants who reached peak VO2 ≥ 18 mL/kg/min (n = 170), who were divided in two groups according to the value of the VE/VCO2 slope (< 35 or ≥ 35). Within this subgroup, those who demonstrated a VE/VCO2 slope ≥ 35 were characterized by shorter 6MWT distance, longer time needed for ‘stand up and go’ test, and significantly lower number of repetitions during the ‘chair stand’ and ‘arm curl’ tests as compared to patients with VE/VCO2< 35.

## 4. Discussion

This is the first study that demonstrates not only physical capacity problems, but also shows reduced physical fitness in patients with HFrEF.

We demonstrated that the decline of aerobic endurance (reflected by shorter distance covered in the 6MWT), limited agility (worse results during the ‘stand up and go’ test) and reduced muscular endurance (assessed in ‘chair stand’ and ‘arm curl’ tests) correlates strongly with reduced peak VO2, shorter exercise time and higher VE/VCO2 slope.

We have shown that there is a relationship between the level of physical fitness and the Weber functional classification. Patients with more advanced HFrEF were characterized by lower values of peak VO2 according to the Weber functional classification as well as by a worse physical fitness as assessed using the Senior Fitness Test.

It is of note that the results of the functional fitness test, a simple non-invasive test that can be performed under any conditions, correlate with the measurement of physical capacity assessed in the CPX. We also demonstrated lower levels of physical fitness in patients with good exercise tolerance (peak VO2 ≥ 18 mL/kg/min) and intensified ventilator response (VE/VCO2 slope ≥ 35). This may point to ventilation disorders and dyspnoea as important factors related to the functionality and mobility of HFrEF patients.

Dyspnoea and fatigue in patients with HF not only occur during prolonged, intensive physical efforts, but also limit the patients’ daily activities. Therefore, our interests focused on the assessment of physical functioning in patients with HF.

An efficient management of HF patients requires precise data derived from appropriate, reproducible, accurate and simple tests, suitable for functional monitoring. This is particularly important in patients with advanced HF, patients qualified for LVAD, TAVI, HTX and patients with other diseases that prevent the CPX test from being performed.

Patients with LVAD report mobility limitations caused by HF conditions long before device implantation. Kitagakii et al. proved a 50% decrease in muscle strength compared to healthy individuals. They also observed nutritional impairment, which combined with reduced physical activity may reduce muscle strength. As a consequence, this leads to muscle atrophy. Moreover, Kazufumi et al. draw attention to the fact that exercise capacity of HF patients is influenced by skeletal muscle dysfunction [22].

Muscle strength (mostly lower and upper limb measured by handgrip, isometric and isokinetic strength testing) is a very important factor in predicting independence in daily living activities in LVAD patients. HF patients (those before LVAD) mostly stay in bed for a long time, which may lead to muscle atrophy. In this study, only 18 out of 38 patients were able to perform a CPX test. This shows that, in this group of patients, cardiac performance is probably the limiting factor during CPX and indicates a need for a different test that could be performed in patients with extreme HF.

Gobbo et al. emphasize that in LVAD (HF patients) the physical function assessment and strength tests should be included. However, isokinetic tests were poorly tolerated, so an alternative should be explored [23].

Hotta et al. examined 75 male CHF patients with chronic kidney disease and suggested that not only estimated glomerular filtration rate but also knee extensor muscle strength may be useful indices for predicting the attainment of an exercise capacity of ≥5 METs in this group of patients [24].

Brunjes at al. provided evidence of lower isometric peak torque and lower total power for knee flexion in patients with advanced, systolic heart failure [25].

The authors’ conclusions are consistent with our assumptions for the need to use simple but reliable methods for assessing functional capacity of patients with advanced, systolic HF.

Examining a group of 31 patients with systolic HF has shown that lower extremity strength (evaluated using a one-repetition maximum (1RM)) is related to peak VO2, VE/VCO2 slope and heart rate [26]. Our observations also confirm worse results during the ‘chair stand’ test, indicating reduced leg muscle endurance, correlated with lower oxygen consumption and ventilator response at the peak of the exercise.

The results presented in this article are concordant with previous studies demonstrating that leg muscle endurance (assessed using the isokinetic dynamometer) was related to peak VO2 and VE/VCO2 slope in 32 patients with HF. Essentially, within the control group, which included six healthy subjects, the correlation with VE/VCO2 slope did not occur. However, this study included a small number of patients representing a relatively limited range of HF advancements (NYHA I and II). Moreover, the assessment was limited to muscular endurance of the lower limbs, measured using a dynamometer, without the functional tests which could be translated to the everyday activity of the patient [27].

We would like to emphasize that the main advantages of the present study are that we examined a relatively high number of patients with HF, as well as that the tests included in the Senior Fitness Test involved not only lower but also upper limbs.

In earlier studies, male patients with HF were divided into two groups according to MET (>5, n = 85 and <5, n = 63). The handgrip and knee extensor/flexor muscle strength was measured using an isokinetic dynamometer. Patients with lower MET demonstrated worse muscle strength as compared to those with higher MET. This study did not involve any functional tests [28]. Moreover, we would like to highlight that our patients were divided into four groups, and that all differences in physical fitness between the groups were significant.

It is possible that the observed relationships are linked to the peripheral HF patho-mechanisms occurring within the skeletal muscles [29,30]. That is why an objective assessment of the physical fitness level from the perspective of time and the dynamics of changes in individual motor skills may be an important element for individual cardiac rehabilitation programs. If so, encouraging patients to perform simple, low-intensity physical exercises (resulting in a slight increase in their fitness) would lead to an improvement of oxygen consumption (reflected by higher peak VO2) or at least an improvement of exercise capacity (in terms of longer exercise duration or lower VE/VCO2 slope without improved peak VO2), especially among patients whose physical fitness is very limited.

Previous studies involving patients with HF demonstrated that maximal inspiratory pressure (MIP) and maximal expiratory pressure (MEP), reflecting the strength of respiratory muscles, are negatively correlated to VE/VCO2 slope [31]. It can be presumed that dyspnoea, excessive ventilation and greater respiratory effort, a manifestation of weakness and fatigue of the respiratory muscles, occur not only at longer-duration exercises (like those during CPX) but also during shorter exercise tasks (e.g., lasting up to 30 s).

The VE/VCO2 slope plays an important role in identifying individuals who are at high risk of both hospitalization and cardiovascular death [32,33]. Hence, in the present study we focused on the physical fitness of patients with excessive ventilatory responses to exercise (i.e., in patients with higher values of VE/VCO2 slope). The results of the analysis conducted among participants with peak VO2 ≥ 18 mL/kg/min, based on comparison with patients who achieved a VE/VCO2 slope ≥ 35, indicated that an extensive ventilatory response to exercise was accompanied by a worse physical fitness level.

Optimal risk stratification assessment still needs development in cardiac patients, especially HF patients. Perone et al. point to the increasingly critical role of cardiovascular imaging in risk assessment. This approach will enable the selection of accurate treatment strategies for patients with increased cardiovascular risk [34]. Stafford et al. measured pulmonary artery wedge pressure (PAWP) using echocardiography. The authors showed that there is a strong correlation between PAWP and cardiovascular deaths or hospitalization in HFrEF patients. Higher PAWP also correlates with exercise capacity; lower peak VO2, high VE/VCO2 slope; shorter 6MWT distance; and lower exercise duration [35].

The results of previous studies indicate that 6MWT could be a useful tool in predicting hospitalization and mortality in systolic and advanced HF patients.

Just as in Pulignano et al., we find that gait speed should be incorporated in the clinical evaluation of HF patients. Furthermore, the authors have proven that gait speed in combination with a validated clinical risk score improves prognostic prediction in patients with HF (EF < 45%) [36].

Patients with HFrEF reach a longer total isovolumic time. Also, a shorter distance in 6MWT is associated with a lower ejection fraction in these patients [37].

Saji et al. examined patients following TAVI, who were assessed by the short physical performance battery (SPPB) and a modified Fried frailty scale. The authors demonstrated that functional capacity assessed by a simple test serves as a prognostic value for rehospitalization. It correlates with frailty, which indicates a need for using short tests that assess functional capacity in a comprehensive assessment of elderly patients with multi-morbidity to qualify for various cardiological and cardiac surgical procedures [38].

The above results prove that patients with HFrEF have a lower functional capacity particularly with aerobic capacity, agility, muscle strength and endurance.

We have emphasized the significance of slope VE/VCO2 and peak VO2 as prognostic factors of rehospitalization and cardiovascular deaths in patients with HF. The results of our study show a high affinity of fitness tests for peak VO2 and slope VE/VCO2.

Taking into consideration other authors’ results, we suggest that for patients who are unable to perform CPX, results of fitness tests may be useful in the context of risk stratification of HFrEF patients.

Results obtained from the Senior Fitness Test, consisting of submaximal tests, during which the patient could freely modulate the pace and speed of exercise, led to a similar conclusion. Patients grouped in each of the four classes differed significantly from responders classified in a higher or lower class in terms of all the elements of the Senior Fitness Test. Consequently, it can be presumed that if the classification proposed by Weber enables us to perform an objective stratification of patients according to the level of their exercise tolerance, cardiac dysfunction and prognosis, then the level of physical fitness would be similarly related to the prognosis or to the risk of HF progression and/or rehospitalization [39].

### Study Limitations

We examined men with HFrEF only, and there are no data regarding either women or remaining strata of LVEF (midrange and preserved). There are no data regarding the length of disease history. Furthermore, we did not include flozins in the pharmacological treatment method due to the fact that they are included in HF treatment guidelines from 2021, and the research group also covers the earlier period.

## 5. Conclusions

Reduced exercise tolerance led to the decline of physical function in patients with HFrEF. As peak VO2 decreases, aerobic capacity, agility, muscle strength and endurance decrease. Moreover, limitations in physical fitness seem to be distinctive for those patients showing excessive ventilatory responses to exercise as reflected by the slope VE/VCO2 ≥ 35 as well as among those whose oxygen consumption was not strongly reduced (peak VO2 ≥ 18 mL/kg/min). These simple tools could be useful for comprehensive functional and clinical status and risk verification in patients with HFrEF, particularly those with extremely low exercise capacity.

## Figures and Tables

**Table 1:** The Senior Fitness Test tasks.

Name of the Test	Methodology of the Test	Result of the Test	Agility
Up-and-go [UG]	The patient is asked to stand up from the chair, walk around a cone located at a distance of 2.44 m and return to the baseline position on the chair, as fast as possible.	The test was performed twice, where the shorter time (measured with a 0.1 s accuracy) was selected for further analyses.	Assessing agility and dynamic balance.
Chair stand	The patient is asked to do as many full stands as possible within 30 s, without the assistance of arms, which remain crossed at the level of the chest.	The number of completed full stands was used as the result of the test.	Assessing the muscular endurance of the lower parts of the body.
Arm curl	The patient is asked to lift 3.5 kg using his forearm as many times as possible within 30 s. In sitting position, the patient was asked to lift weights by flexion and pronation of the forearm of the dominant hand, followed by return to the baseline.	The total number of arm curls was used as the result of the test.	Assessing upper body muscular endurance.
Sit-and-reach [SR]	The patient is asked to reach the toes using hands, while sitting at the edge of a chair with the dominant leg extended and the foot resting on the floor (dorsiflexion).	The test was performed twice and the shorter distance between the fingers and toes (recorded with a 0.5 cm accuracy) was selected for further analyses.	Assessing the flexibility of the lower parts of the body.
Back scratch [BS]	The subject tries to reach the hand held at the middle of the back using the other hand moved over the shoulder and, if possible, to overlap the fingers.	The test was performed twice and the shorter distance between the straightened fingers of both hands (measured with a 0.5 cm accuracy) was selected for further analyses.	Assessing the flexibility of the upper parts of the body.
Six-minute walk test [6MWT]	The patient has to walk back and forth along the 30 m hospital corridor as fast as possible in order to cover the longest possible distance for 6 min.	The distance recorded with a one-meter accuracy is used as the result of this test.	Assessing aerobic capacity.

**Table 2:** Baseline clinical characteristic of men with HFrEF.

Variables	Men with HFrEF (n = 382)	Weber Class D (n = 37)	Weber Class C (n = 132)	Weber Class B (n = 120)	Weber Class A (n = 93)
Age, years	61 ± 10	63.1 ± 8.3	63.6 ± 9.6	61.1 ± 9.6	54.9 ± 9.3
BMI, kg/m^2^	27.9 ± 4.5	27.4 ± 4.8	28.3 ± 4.3	28.2 ± 4.7	26.4 ± 4.4
CHF aetiology, ischemic %	249 (65%)	28 (76%)	100 (73%)	75 (62%)	51 (56%)
NYHA class, I/II/III/IV	63/190/123/6 (16/50/32/2%)	0/7/26/4 (0/19/70/11%)	4/73/53/2 (3/55/40/2%)	24/69/27/0 (20/57/23/0%)	35/51/7/0 (37/55/8/0%)
LVEF, %	30.5 ± 8.3	26.5 ± 8.5	29.7 ± 7.8	31.0 ± 8.6	32.5 ± 7.9
NT-pro BNP [pg/mL]	2334 ± 3652	5705.8 ± 6299.8	2748 ± 4012	1971 ± 2285	774 ± 1287
Ex-time [min]	10.9 (7.5–12.8)	4.8 (4.0–6.5)	8.5 (6.4–10.3)	11.8 (10.3–12.7)	14.4 (12.7–15.9)
Peak VO2 [ml/kg/min.]	16.5 (13.3–20.0)	9.3 (7.6–9.6)	13.6 (12.2–14.7)	17.9 (16.8–18.8)	22.8 (21.8–25.6)
Slope VE/VCO2	35.7 (30.7–42.3)	50.0 (37.5–61.2)	38.0 (32.3–45.6)	35.2 (30.1–39.9)	31.6 (27.3–36.4)
Diabetes mellitus n, (%)	100 (26%)	15 (40.5%)	46 (34.8%)	26 (21.7%)	13 (14.0%)
Atrial fibrillation n, (%)	104 (27%)	15 (40.5%)	40 (30.3%)	40 (33.3%)	9 (9.7%)
Haemoglobin, g/dL	14.3 ± 1.4	13.5 ± 1.8	14.1 ± 1.4	14.3 ± 1.5	14.7 ± 1.1
Treatment:					
ACE-I and/or ARB n, (%)	352 (92%)	32 (86.5%)	122 (92.4%)	111 (92.5%)	87 (93.5%)
Beta-blockers	364 (95%)	35 (94.6%)	127 (96.2%)	114 (95.0%)	88 (94.6%)
Digoxin	105 (27%)	16 (43.2%)	38 (28.8%)	35 (29.2%)	16 (17.2%)
Statins	297 (77%)	29 (78.4%)	104 (78.8%)	94 (78.3%)	70 (75.3%)
Aldosterone receptor antagonists	176 (46%)	21 (56.6%)	71 (53.8%)	46 (38.3%)	38 (40.9%)
Loop diuretics	319 (83%)	35 (94.6%)	119 (90.2%)	98 (81.7%)	67 (72.0%)

CHF, chronic heart failure; BMI, body mass index; NYHA, New York Heart Association; LVEF, left ventricular ejection fraction; NT-proBNP, N-terminal pro-I type natriuretic peptide; ACE-I angiotensin-converting enzyme inhibitor; ARB angiotensin receptor blocker. Data are presented as means ± standard deviation, medians (with lower and upper quartiles) or numbers (with percentages) where appropriate.

**Table 3:** The relationship between physical fitness and exercise capacity in patients with HF.

The Senior Fitness Test	Ex-Time [min]	Peak VO2 [mL/kg/min]	Slope VE/VCO2
	Spearman Correlation Coefficients		
6 min walk test [m]	0.57 *	0.51 *	−0.35 *
Stand up and go [s]	−0.46 *	−0.43 *	0.27 *
Chair stand [number]	0.45 *	0.41 *	−0.28 *
Arm curl [number]	0.40 *	0.45 *	−0.29 *
Chair sit and reach [cm]	0.04	0.02	0.04
Back scratch [cm]	0.10	0.11	0.05

* *p* < 0.05.

**Table 4:** The comparison of physical fitness between patients with heart failure divided according to Weber’s classification, reflecting 4 levels of deterioration of exercise capacity.

The Senior Fitness Test	Weber Class D N = 37	Weber Class C N = 132	Weber Class B N = 120	Weber Class A N = 93	Weber Class D vs. Weber Class C	Weber Class C vs. Weber Class B	Weber Class B vs. Weber Class A
6 min walk test [m]	336 (270–405)	450 (398–500)	504 (450–562)	540 (480–605)	*p* < 0.05	*p* < 0.0001	*p* < 0.0001
Stand up and go [s]	7.0 (6.0–8.1)	5.8 (5.1–6.7)	5.4 (4.8–5.9)	4.8 (4.3–5.3)	*p* < 0.001	ns	*p* < 0.05
Chair stand [number]	10 (9.8–12)	13 (11–16)	14 (13–17)	16 (14–19)	*p* < 0.05	*p* < 0.0001	*p* < 0.0001
Arm curl [number]	10.5 (9–13)	14 (12–17)	16 (13–19)	18 (15–21)	*p* < 0.001	*p* < 0.0001	*p* < 0.0001
Chair sit and reach [cm]	−4.5 (−11.0–0.0)	0.0 (−8.0–7.0)	0.0 (−7.0–5.0)	0.0 (−8.0–3.5)	ns	ns	ns
Back scratch [cm]	−9.0 (−21.0 = 0.0)	−9.0 (−18.0–0.0)	−7.5 (−13.0–0.0)	−4.5 (−11.0–2.0)	ns	ns	ns

**Table 5:** Physical fitness in patients with preserved exercise capacity and VE/VCO2 slope ≤ 35 and VE/VCO2 slope > 35.

The Senior Fitness Test	Peak VO2 > 18 mL/kg/min and VE/VCO2 Slope ≤ 35 Median (Upper and Lower Quartile) N = 90	Peak VO2 > 18 mL/kg/min and VE/VCO2 Slope > 35 Median (Upper and Lower Quartile) N = 67	*p* Value
6 min walk test [m]	550 (490–601)	499 (456–547)	*p* < 0.0001
Stand up and go [s]	4.8 (4.3–5.5)	5.3 (4.7–5.9)	*p* < 0.0001
Chair stand [number]	17 (14.0–18.0)	14.0 (12.5–16.0)	*p* < 0.0001
Arm curl [number]	19 (16.0–21.0)	15.0 (13.0–18.0)	*p* < 0.00001
Chair sit and reach [cm]	0.0 (−7.5–5.5)	0.0 (−8.0–3.0)	*p* = 0.96
Back scratch [cm]	−7.0 (−12.0–2.0)	−4.0 (−13.0–2.0)	*p* = 0.95

## Data Availability

The datasets analysed during the current study are available from the corresponding author upon reasonable request.

## References

[1] McDonagh T.A., Metra M., Adamo M., Gardner R.S., Baumbach A., Böhm M., Burri H., Butler J., Čelutkienė J., Chioncel O. (2023). 2023 Focused Update of the 2021 ESC Guidelines for the diagnosis and treatment of acute and chronic heart failure. Eur. Heart J..

[2] McDonagh T.A., Metra M., Adamo M., Gardner R.S., Baumbach A., Böhm M., Burri H., Butler J., Čelutkienė J., Chioncel O. (2021). 2021 ESC Guidelines for the diagnosis and treatment of acute and chronic heart failure. Eur. Heart J..

[3] Bauersachs J., Soltani S. (2024). Herzinsuffizienz: Leitlinien-Update der ESC 2023 [Heart failure: Update of the ESC 2023 guidelines]. Herz.

[4] Pardaens S., Calders P., Derom E., De Sutter J. (2013). Exercise intolerance in heart failure: Update on exercise parameters for diagnosis, prognosis and therapeutic interventions. Acta Cardiol..

[5] Dubé B.P., Agostoni P., Laveneziana P. (2016). Exertional dyspnoea in chronic heart failure: The role of the lung and respiratory mechanical factors. Eur. Respir. Rev..

[6] Wegrzynowska-Teodorczyk K., Rudzińska E., Jankowska E., Grzesło A., Nowakowska K., Lazorczyk M., Banasiak W., Ponikowski P., Woźniewski M. (2010). Determinants of physical fitness in males with systolic heart failure. Kardiol. Pol..

[7] Myers J., Arena R., Cahalin L.P., Labate V., Guazzi M. (2015). Cardiopulmonary Exercise Testing in Heart Failure. Curr. Probl. Cardiol..

[8] Schefold J.C., Filippatos G., Hasenfuss G., Anker S.D., von Haehling S. (2016). Heart failure and kidney dysfunction: Epidemiology, mechanisms and management. Nat. Rev. Nephrol..

[9] Kato A. (2013). Muscle wasting is associated with reduced exercise capacity and advanced disease in patients with chronic heart failure. Future Cardiol..

[10] Delgado B.M., Lopes I., Gomes B., Novo A. (2020). Early rehabilitation in cardiology—Heart failure: The ERIC-HF protocol, a novel intervention to decompensated heart failure patients rehabilitation. Eur. J. Cardiovasc. Nurs..

[11] Forman D.E., Fleg J.L., Kitzman D.W., Brawner C.A., Swank A.M., McKelvie R.S., Clare R.M., Ellis S.J., Dunlap M.E., Bittner V. (2012). 6-min walk test provides prognostic utility comparable to cardiopulmonary exercise testing in ambulatory outpatients with systolic heart failure. J. Am. Coll. Cardiol..

[12] Tumminello G., Guazzi M., Lancellotti P., Piérard L.A. (2007). Exercise ventilation inefficiency in heart failure: Patho physiological and clinical significance. Eur. Heart J..

[13] Fu T.C., Yang N.I., Wang C.H., Cherng W.-J., Chou S.-L., Pan T.-L., Wang J.-S. (2016). Aerobic interval training elicits different heodynamic adaptation between heart failure patients with preserved and reduced eject fraction. Am. J. Phys. Med. Rehabil..

[14] Canada J.M., Trankle C.R., Buckley L.F., Carbone S., Abouzaki N.A., Kadariya D., Shah K., Cooke R., Kontos M.C., Patel J. (2017). Severely impaired cardiorespiratory fitness in patients with recently decompensated systolic heart failure. Am. J. Cardiol..

[15] Brawner C.A., Shafiq A., Aldred H.A., Ehrman J.K., Leifer E.S., Selektor Y., Tita C., Velez M., Williams C.T., Schairer J.R. (2015). Comprehensive analysis of cardiopulmonary exercise testing and mortality in patients with systolic heart failure: The Henry Ford Hospital cardiopulmonary exercise testing (FIT-CPX) project. J. Card. Fail..

[16] Keteyian S.J., Patel M., Kraus W.E., Brawner C.A., McConnell T.R., Piña I.L., Leifer E.S., Fleg J.L., Blackburn G., Fonarow G.C. (2016). (HF-ACTION Investigators). Variables Measured During Cardiopulmonary Exercise Testing as Predictors of Mortality in Chronic Systolic Heart Failure. J. Am. Coll. Cardiol..

[17] Dosbaba F., Žurková P., Baťalík L., Dosbaba H., Felšőci M., Vysoký R., Špinar J., Ludka O. (2017). The importance of evaluating the effectiveness of the ventilation VE/VCO2 slope in patients with heart failure. Vnitr. Lek. Winter.

[18] Rikli R.E., Jones C.J. (1999). Development and validation of a functional fitness test for community-residing older adults. J. Aging Phys. Act..

[19] Guazzi M., Adams V., Conraads V. (2012). (EACPR/AHA Joint Scientific Statement). Clinical recommendations for cardiopulmonary exercise testing data assessment in specific patient populations. Circulation.

[20] Soumagne D. (2012). Weber classification in cardiac rehabilitation. Acta Cardiol..

[21] Węgrzynowska-Teodorczyk K., Dąbrowska E., Jankowska E.A., Ponikowski P., Banasiak W., Woźniewski M. (2010). Usefulness of the Fullerton test in assessment of physical fitness in men with stable heart failure. Adv. Rehabil..

[22] Kitagaki K., Aoki T., Miura H., Shimada Y., Konishi H., Tsukamoto Y., Noguchi T. (2024). Depressive symptoms, right ventricular function, and muscular strength are associated with peak oxygen uptake in patients with implantable left ventricular assist devices. Artif. Organs..

[23] Gobbo S., Favro F., Bullo V., Cugusi L., Blasio A.D., Bortoletto A., Bocalini D.S., Gasperetti A., Ermolao A., Bergamin M. (2022). Muscle strength, aerobic capacity, and exercise tolerance are impaired in left ventricular assist devices recipients: A pilot study. Front. Physiol..

[24] Hotta C., Hiraki K., Watanabe S., Izawa K.P., Yasuda T., Osada N., Omiya K., Kimura K. (2014). Knee extensor muscle strength and index of renal function associated with an exercise capacity of 5 metabolic equivalents in male chronic heart failure patients with chronic kidney disease. Clin. Exp. Nephrol..

[25] Brunjes D.L., Dunlop M., Wu C., Jones M., Kato T.S., Kennel P.J., Armstrong H.F., Choo T.H., Bartles M.N., Forman D.E. (2016). Analysis of Skeletal Muscle Torque Capacity and Circulating Ceramides in Patients with Advanced Heart Failure. J. Card. Fail..

[26] Zavin A., Arena R., Joseph J., Allsup K., Daniels K., Schulze P.C., Lecker S., E Forman D. (2013). Dynamic assessment of ventilatory efficiency during recovery from peak exercise to enhance cardiopulmonary exercise testing. Eur. J. Prev. Cardiol..

[27] Saval M.A., Kerrigan D.J., Ophaug K.M., Ehrman J.K., Keteyian S.J. (2010). Relationship between leg muscle endurance and (.)VE/(.)VCO2 slope in patients with heart failure. J. Cardiopulm. Rehabil. Prev..

[28] Izawa K.P., Watanabe S., Oka K., Hiraki K., Morio Y., Kasahara Y., Watanabe Y., Katata H., Osada N., Omiya K. (2012). Upper and lower extremity muscle strength levels associated with an exercise capacity of 5 metabolic equivalents in male patients with heart failure. J. Cardiopulm. Rehabil. Prev..

[29] Cicoira M. (2002). Muscle changes and exercise intolerance in congestive heart failure: Main role of the periphery. Ital. Heart J. Suppl..

[30] Piepoli M.F., Coats A.J. (2013). The ‘skeletal muscle hypothesis in heart failure’ revised. Eur. Heart J..

[31] Kasahara Y., Izawa K.P., Watanabe S., Osada N., Omiya K. (2015). The Relation of Respiratory Muscle Strength to Disease Severity and Abnormal Ventilation During Exercise in Chronic Heart Failure Patients. Res. Cardiovasc. Med..

[32] Poggio R., Arazi H.C., Giorgi M., Miriuka S.G. (2010). Prediction of severe cardiovascular events by VE/VCO2 slope versus peak VO2 in systolic heart failure: A meta-analysis of the published literature. Am. Heart J..

[33] Shen Y., Zhang X., Ma W., Song H., Gong Z., Wang Q., Che L., Xu W., Jiang J., Xu J. (2015). VE/VCO2 slope and its prognostic value in patients with chronic heart failure. Exp. Ther. Med..

[34] Perone F., Bernardi M., Redheuil A., Mafrica D., Conte E., Spadafora L., Ecarnot F., Tokgozoglu L., Santos-Gallego C.G., Kaiser S.E. (2023). Role of Cardiovascular Imaging in Risk Assessment Recent Advances, Gaps in Evidence and Future Directions. J. Clin. Med..

[35] Stafford P.L., Purvis A., Bilchick K., Nguyen J.D.K., Patil P., Baldeo C., Mehta K., Kwon Y., Breathett K., Shisler D. (2024). Echocardiographic derived pulmonary artery wedge pressure is associated with mortality, heart hospitalizations and functional capacity in chronic systolic heart failure: Insights from the HF-ACTION trial. J. Echocardiogr..

[36] Pulignano G., Del S.D., Di L.A., Alunni G., Senni M., Tarantini L., Cioffi G., Tinti M.D., Barbati G., Minardi G. (2016). Incremental Value of Gait Speed in Predicting Prognosis of Older Adults With Heart Failure: Insights From the IMAGE-HF Study. JACC Heart Fail..

[37] Gardin J.M., Leifer E.S., Fleg J.L., Whellan D., Kokkinos P., LeBlanc M.H., Wolfel E., Kitzman D.W. (2009). HF-ACTION Investigators. Relationship of Doppler-Echocardiographic left ventricular diastolic function to exercise performance in systolic heart failure: The HF-ACTION study. Am. Heart J..

[38] Saji M., Higuchi R., Tobaru T., Iguchi N., Takanashi S., Takayama M., Isobe M. (2018). Impact of Frailty Markers for Unplanned Hospital Readmission Following Transcatheter Aortic Valve Implantation. Circ. J..

[39] Guazzi M., Myers J., Abella J., Peberdy M.A., Bensimhon D., Chase P., Arena R. (2008). The added prognostic value of ventilatory efficiency to the Weber classification system in patients with heart failure. Int. J. Cardiol..

